# Clinical Usefulness of Monitoring Expression Levels of* CCL24* (Eotaxin-2) mRNA on the Ocular Surface in Patients with Vernal Keratoconjunctivitis and Atopic Keratoconjunctivitis

**DOI:** 10.1155/2016/3573142

**Published:** 2016-09-18

**Authors:** Yukiko Shiraki, Jun Shoji, Noriko Inada

**Affiliations:** Division of Ophthalmology, Department of Visual Sciences, Nihon University School of Medicine, Tokyo, Japan

## Abstract

*Purpose*. This study aimed to evaluate the clinical efficacy of using expression levels of* CCL24* (eotaxin-2) mRNA on the ocular surface as a biomarker in patients with vernal keratoconjunctivitis (VKC) and atopic keratoconjunctivitis (AKC).* Methods*. Eighteen patients with VKC or AKC (VKC/AKC group) and 12 control subjects (control group) were enrolled in this study. The VKC/AKC clinical score was determined by objective findings in patients by using the 5-5-5 exacerbation grading scale. All subjects underwent modified impression cytology and specimens were obtained from the upper tarsal conjunctiva. Expression levels of* CCL24* (eotaxin-2) mRNA on the ocular surface were determined using real-time reverse transcription polymerase chain reaction.* Results*. The VKC group was divided into two subgroups, depending on the clinical score: the active stage subgroup with 100 points or more of clinical scores and the stable stage subgroup with 100 points or less.* CCL24* (eotaxin-2) mRNA expression levels in the active VKC/AKC stage subgroup were significantly higher than those in the stable VKC/AKC subgroup and the control group. Clinical scores correlated significantly with* CCL24* (eotaxin-2) mRNA expression levels in the VKC group.* Conclusions*.* CCL24* (eotaxin-2) mRNA expression levels on the ocular surface are a useful biomarker for clinical severity of VKC/AKC.

## 1. Introduction

Allergic conjunctival diseases (ACD) are conjunctival inflammatory disorders, characterized by an immediate hypersensitivity reaction associated with antigen-specific IgE antibodies [1]. Patients with ACD are usually categorized into the following types based on clinical criteria: seasonal allergic conjunctivitis (SAC), perennial allergic conjunctivitis (PAC), vernal keratoconjunctivitis (VKC), atopic keratoconjunctivitis (AKC), and giant papillary conjunctivitis (GPC) [2]. SAC and PAC are characterized by conjunctival hyperemia, conjunctival edema, and papillary hyperplasia of tarsal conjunctiva and are classified as mild ACD. In contrast, VKC is characterized by the development of proliferative lesions of the conjunctiva, including giant papillary proliferation of the tarsal conjunctiva and gelatinous cell infiltration of the limbal conjunctiva. VKC also complicates corneal disorders, such as shield ulcer and punctate corneal keratitis, and is classified as a severe ACD, because the visual prognosis may be poor. AKC usually develops in older patients with atopic dermatitis and also complicates severe ocular surface diseases, including giant papillary conjunctivitis, shield ulcer, and dry eye.

In shield ulcers of patients with VKC and AKC, depositions of major basic protein and eosinophil cationic protein (ECP), which comprise specific granules of eosinophils, have reportedly been observed histologically in the corneal ulcerative lesions [3, 4]. Furthermore, ECP levels in the tears of VKC and AKC patients are reportedly increased, in comparison with those in controls [5–7]. Therefore, the pathophysiological characteristics of these severe ACD, including VKC and AKC, include eosinophilic inflammation in the conjunctiva.

Eotaxin is a member of the CC chemokine family and is divided into three subfamilies, namely, CCL11/eotaxin-1, CCL24/eotaxin-2, and CCL26/eotaxin-3. Eotaxin-1, eotaxin-2, and eotaxin-3 interact with the CC chemokine receptor 3 (CCR3). Representative inflammatory cells expressing CCR3 on their cell surfaces are eosinophils, type-2 helper T cells (Th2), and basophils. IL-13, which is a Th2-derived cytokine, induces eotaxin in vitro through activation of the IL-4R*α* receptor/STAT-6 pathway [8, 9]. Therefore, eotaxin-1, eotaxin-2, and eotaxin-3 are thought to be allergic inflammation-related chemokines.

In the eotaxin subfamily, it has been reported that increased eotaxin-2 levels in tears and expression of* CCL24* (eotaxin-2) mRNA on the ocular surface are more common in VKC patients than those of eotaxin-1 and eotaxin-3 [10, 11]. However, the relationship between the expression levels of* CCL24* (eotaxin-2) mRNA on the ocular surface and the severity of severe ACD, including VKC and AKC, has not been fully investigated.

In this study, we evaluated the clinical efficacy of using* CCL24* (eotaxin-2) mRNA expression levels on the ocular surface in patients with severe ACD, including VKC and AKC, as a biomarker for severe ACD.

## 2. Subjects and Methods

This study was approved by the institutional review board of the Nihon University School of Medicine and adhered to the tenets of the Declaration of Helsinki. Informed consent was obtained from all study subjects.

### 2.1. Subjects

This study included 18 consecutive patients with VKC or AKC treated at the Department of Ophthalmology, Nihon University Itabashi Hospital, Tokyo, Japan, from August 2012 to January 2015 (AKC/VKC group), and 12 healthy volunteers who did not have any personal or family history of atopic disease, were not affected by ocular surface diseases, or have a history of wearing contact lenses as controls (control group). Demographic data of the subjects are shown in Table 1. Objective diagnoses were made in VKC and AKC patients by means of slit-lamp clinical examination and serum examination for antigen-specific IgE antibodies, according to the Japanese guidelines for ACD [2]. Patients with ocular surface disease other than ACD, including lagophthalmos, blepharospasm, conjunctival chalasis, dry eye, infectious conjunctivitis, infectious keratitis, Stevens-Johnson syndrome, and ocular pemphigoid, were excluded. Nontreated patients or patients treated with antiallergic ophthalmic solutions alone, such as mast cell stabilizers, histamine H_1_ receptor antagonists, corticosteroids, and immunosuppressive agents, were included in the study. Patients who used oral medicines or injections for treating allergic diseases and those who received immunotherapy were excluded from the study.

### 2.2. Clinical Score Grading

Clinical scores of objective findings in the AKC/VKC group were determined using the 5-5-5 exacerbation grading scale for ACD [12]. The AKC/VKC group was divided into two subgroups depending on the clinical score: the active stage subgroup with clinical scores of 100 points or more (*n* = 6) and the stable stage subgroup with 100 points or less (*n* = 12).

### 2.3. Modified Impression Cytology

Modified impression cytology was performed after instillation of topical oxybuprocaine 0.4% (Benoxil, Santen, Osaka, Japan). Schirmer's test strips (Schirmer Tear Production Measuring Strips, Showa Yakuhin Kako, Tokyo, Japan) were applied to the upper tarsal conjunctiva, pressed gently using a glass rod, and then removed. The membrane was preserved in RNAlater RNA Stabilization Reagent (Qiagen, Hilden, Germany) until analysis.

### 2.4. Real-Time Reverse Transcription Polymerase Chain Reaction

Total RNA was harvested from each Schirmer tested paper using an RNeasy® Mini Kit (QIAGEN, Hilden, Germany) following the manufacturer's instructions. cDNA was then synthesized using a High-Capacity cDNA Reverse Transcription Kit (Life Technologies Japan, Tokyo, Japan), according to the manufacturer's instructions.

To detect expression of* CCL24* (eotaxin-2) mRNA, real-time reverse transcription polymerase chain reaction (real-time RT-PCR) was performed using a commercial PCR master mix (TaqMan Universal PCR Master Mix; Life Technologies, Tokyo, Japan) and predesigned primers (Life Technologies) for* CCL24* (Eotaxin-2; Hs00171082_m1). Samples were analyzed using the Step One Plus*™* real-time PCR system (Life Technologies) and comparative threshold (Ct) values were obtained. Target Ct values were normalized to those of* GAPDH* (Hs99999905_m1) in the same sample. Data were analyzed using the ΔΔCt method.

### 2.5. Statistical Analysis

Differences between AKC/VKC and control groups were identified using Welch's *t*-test or the chi-square test. The results for* CCL24* (eotaxin-2) mRNA expression on the ocular surface were evaluated using the nonparametric Steel-Dwass test. Spearman's rank correlation coefficient was used to evaluate whether* CCL24* (eotaxin-2) mRNA expression correlated with the clinical score. A *P* value of <0.05 was regarded as statistically significant.

## 3. Results

### 3.1. Expression Levels of Eotaxin-2 mRNA on Ocular Surface

For* CCL24* (eotaxin-2) mRNA expression on the ocular surface, 6 of 6 patients in the active stage subgroup of AKC/VKC group were* CCL24-* (eotaxin-2-) positive, with median (range) levels of 133 (27.6–232). Eleven of 12 patients in the stable stage subgroup of AKC/VKC group were* CCL24-* (eotaxin-2-) positive, with median (range) levels of 5.99 (0.140–33.2); the remaining patient had levels below the lower limit of detection. Eight of 12 patients in the control group were* CCL24-* (eotaxin-2-) positive, with median (range) levels of 0.98 (0.08–20.2), while 4 patients had levels below the lower limit of detection.

**Figure 1:**
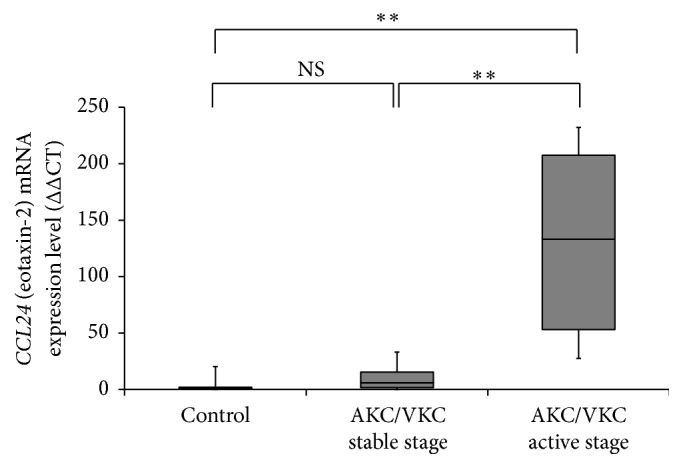
The levels of* CCL24* (eotaxin-2) mRNA expression on the ocular surface in AKC/VKC and control group. The levels of* CCL24* (eotaxin-2) mRNA expression on the ocular surface in the active stage subgroup of the AKC/VKC group were significantly higher than those in the stable stage subgroup of AKC/VKC group and the control group (*P* < 0.01, *P* < 0.01, respectively, Steel-Dwass test). ^*∗*^ *p* < 0.05; NS: not significant.

The expression levels of* CCL24* (eotaxin-2) mRNA on the ocular surface were significantly higher in the active stage than in the stable stage AKC/VKC subgroup and the control group (both* P* < 0.01; Steel-Dwass test; Figure 1).

### 3.2. Relationship between Clinical Severity and Expression Levels of* CCL24* (Eotaxin-2) mRNA

**Figure 2:**
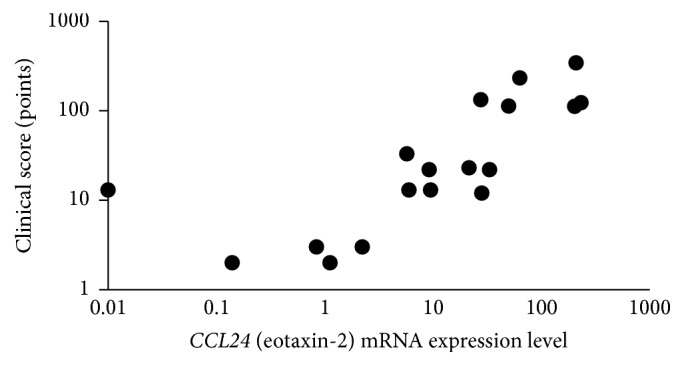
Comparison of clinical scores and levels of* CCL24* (eotaxin-2) mRNA expression on the ocular surface. In the patients with AKC/VKC, clinical scores were significantly correlated with the levels of* CCL24* (eotaxin-2) mRNA expression on the ocular surface (*ρ* = 0.795, *P* < 0.01, Spearman's rank correlation coefficient).

The median value (range) of the clinical scores in the active and stable stage subgroups of AKC/VKC group were 13 (2–33) and 128 (343–112), respectively. In patients with AKC/VKC, clinical scores were significantly correlated with the levels of (*CCL24*) eotaxin-2 mRNA expression on the ocular surface (*ρ* = 0.795,* P* < 0.01, Spearman's rank correlation coefficient; Figure 2).

### 3.3. Case Presentation

#### 3.3.1. Patient 1

**Figure 3:**
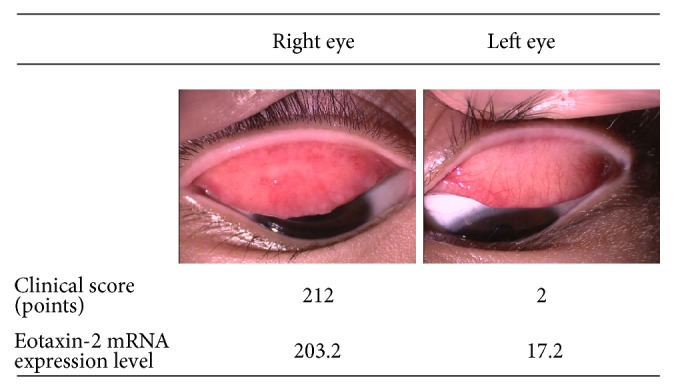
Comparison of the* CCL24* (eotaxin-2) mRNA expression in bilateral eyes of a vernal keratoconjunctivitis (VKC) patient. A 10-year-old boy had VKC, showing laterality in the severity of the objective findings. The levels of* CCL24* (eotaxin-2) mRNA expression on the ocular surface were low (17.2) in the left eye with mild VKC and high (203.2) in the right eye with severe VKC.

A 10-year-old boy was diagnosed as having VKC and had been under treatment for VKC by a local doctor for 6 years. His clinical observation of VKC repeated exacerbation and remission, and his severity of VKC was different in right and left active giant papillae and exfoliative epithelial keratopathy was present in his right eye; his clinical score was 212, and his* CCL24* (eotaxin-2) mRNA expression levels on the ocular surface were 203.2. In his left eye, papillary lesions and hyperemia at the upper palpebral conjunctiva were observed, and the clinical score was 2, while the* CCL24* (eotaxin-2) mRNA expression level on the ocular surface was 17.2 (Figure 3).

#### 3.3.2. Patient 2

**Figure 4:**
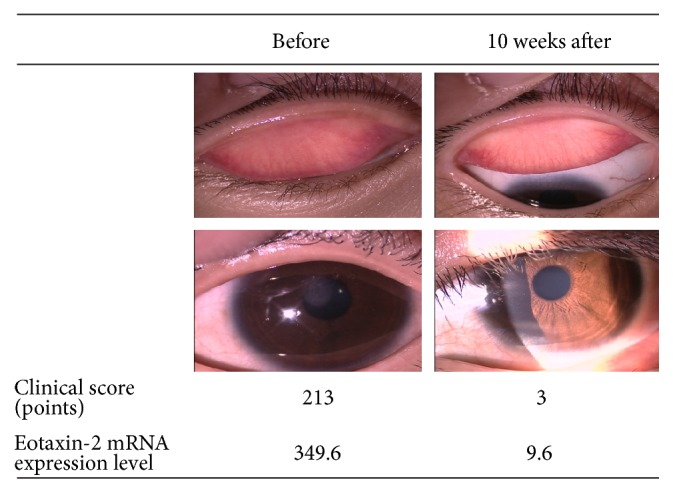
Comparison of* CCL24* (eotaxin-2) mRNA expression levels before and after treatment. A 10-year-old girl with vernal keratoconjunctivitis (VKC) was treated with tacrolimus ophthalmic suspension and disodium cromoglicate ophthalmic solution. With this treatment, her clinical score and levels of* CCL24* (eotaxin-2) mRNA expression decreased, as compared to those before treatment.

A 10-year-old girl was diagnosed as having VKC and had been under treatment for VKC by a doctor for 1 year. She experienced pain in her right eye and also had recurrence of corneal plaque and was referred to our hospital. At her first examination, she demonstrated shield ulcer in her right eye, giant papillae, and palpebral conjunctiva with a velvety appearance. Topical 0.1% tacrolimus ophthalmic suspension (Talymus® Ophthalmic Suspension 0.1%, Senju Pharmaceutical, Co., Ltd., Osaka, Japan) and 2% sodium cromoglicate ophthalmic solution (Intal® Ophthalmic Solution 2%, Sanofi, Tokyo, Japan) were administered. At commencement of treatment with tacrolimus ophthalmic suspension, her clinical score was 213 and the level of* CCL24* (eotaxin-2) mRNA expression on the ocular surface was 349.6. During the first 10 weeks of treatment, the shield ulcer was relieved and the giant papillae disappeared. At this point, the clinical score was 3 points and the* CCL24* (eotaxin-2) mRNA expression level on the ocular surface was 9.6 (Figure 4).

## 4. Discussion

In this study, we assessed the usefulness of* CCL24* (eotaxin-2) mRNA expression levels on the ocular surface as a biomarker of the severity of ACD. We found that these levels correlated well with the clinical score reflecting objective findings in patients with AKC and VKC. As a method for sampling the ocular surface to test the expression levels of* CCL24* (eotaxin-2) mRNA, we used a modified impression cytology method. This method entailed a membrane biopsy technique, as used for impression cytology, but used filter paper instead of nitrocellulose membrane for the biopsy. In the conventional impression cytology method, the specimen obtained using a nitrocellulose membrane is examined histologically. However, the major concern of this method is the ocular sensation of a foreign body and the ocular pain experienced after the examination; repeated examinations for follow-up of the biomarker may cause discomfort for the patients. Changing to the filter paper for impression cytology sampling and using a quantitative method (real-time RT-PCR) allowed assessment of the biomarker on the ocular surface. The specimens obtained by modified impression cytology most likely included conjunctival epithelial and invading inflammatory cells, in addition to tears and mucin. It was therefore considered useful for investigating inflammation-associated factors expressed by conjunctival epithelial cells and inflammatory cells as potential biomarkers of allergic inflammation at the ocular surface.

In this study, we elucidated a significant correlation between clinical observations and CCL24 expression on the ocular surface of AKC/VKC patients with or without treatment with ophthalmic solutions. These results suggest that CCL24 expression on the ocular surface is suitable as a biomarker of severe allergic conjunctival diseases. In previous reports, it has been demonstrated that eotaxin-1 plays a critical role in eosinophilic infiltration in the conjunctiva and cornea of patients with ACD [10, 11, 13, 14]. However, the concentration of eotaxin-2 in tears has been reported to be higher than those of eotaxin-1 in ACD patients [10]. Therefore, in this study, we investigated the usefulness of* CCL24* (eotaxin-2) mRNA expressed on the ocular surface as a biomarker for patients with AKC/VKC. We found that expression levels of* CCL24* (eotaxin-2) mRNA on the ocular surface were significantly increased and correlated with the clinical score in the active stage of VKC.

Eotaxin-1 is reportedly produced by corneal keratinocytes [13, 14], conjunctival fibroblasts [11], CD68-positive cells in the conjunctiva, and eosinophils in the conjunctiva [15]. However, the cellular source of eotaxin-2 in tears was not fully understood. Previously, we have reported that the tear levels of eotaxin-2 correlated significantly with those of eosinophil cationic protein and that epithelial cells in conjunctival smears of patients with VKC expressed eotaxin-2, based on immunohistochemistry [10]. Leonardi and colleagues [11] reported that tear levels of eotaxin-1 and eotaxin-2 significantly correlated with the percentage of eosinophils in tears. Therefore, conjunctival epithelial cells and eosinophils are thought to be candidate eotaxin-2-producing cells on the ocular surface. Furthermore, expression levels of* CCL24* (eotaxin-2) mRNA in modified impression cytology may be a good biomarker for evaluating allergic inflammation in the conjunctivas of patients with AKC/VKC.

In these case reports, we showed differences between the right and left eye in the severity of VKC based on the quantitative analysis of* CCL24* (eotaxin-2) mRNA levels. Furthermore, in the 10-year-old girl with VKC, we were able to show the therapeutic effect of treatment by tacrolimus instillation by the reduction of both clinical scores and expression levels of* CCL24* (eotaxin-2) mRNA. Therefore, in AKC/VKC patients undergoing treatment, monitoring of the expression levels of* CCL24* (eotaxin-2) mRNA may provide a useful index of exacerbation and therapeutic response.

The limitation of this study included the small sample size, and a lack of patients with mild ACD, such as SAC and PAC. Further investigation is necessary for verifying the usefulness of* CCL24* (eotaxin-2) mRNA levels as a biomarker of ACD in a large sample that includes patients with SAC and PAC. Another limitation of this study was that AKC/VKC patients not receiving treatment and those only receiving treatment with ophthalmic solutions were enrolled. The* CCL24* mRNA expression on the ocular surface may be affected by treatment with ophthalmic solutions. However, the efficacy of the therapeutic agent is one of the items measured by a biomarker. Therefore, further investigation on the therapeutic effect of antiallergic treatment in a large cohort, including untreated patients with allergic conjunctival diseases, using eotaxin-2 expression as a biomarker, will be necessary in the future.

## 5. Conclusion

Expression levels of* CCL24* (eotaxin-2) mRNA on the ocular surface are a useful biomarker of the clinical severity of AKC/VKC.

## Figures and Tables

**Table 1:** Subjects and their demographic data.

	Control	AKC/VKC	*P* value
Number of subjects	12	18	
Age (years) (mean ± SD)	25.5 ± 2.07	21.9 ± 13.1	0.272
Gender (male : female)	10 : 2	14 : 4	0.709
Positive ratio of *CCL24* (eotaxin-2) mRNA expression			
Positive	8	17	**0.046**
Negative	4	1	

*AKC*: atopic keratoconjunctivitis; *VKC*: vernal keratoconjunctivitis.

Negative: below the lowest level of detection limit of real-time reversed transcription polymerase chain reaction.
